# Absolute Configuration of the New 3-*epi*-cladocroic Acid from the Mediterranean Sponge *Haliclona Fulva*

**DOI:** 10.3390/metabo3010024

**Published:** 2013-01-14

**Authors:** Grégory Genta-Jouve, Olivier P. Thomas

**Affiliations:** Nice Institute of Chemistry UMR 7272 CNRS-PCRE, University of Nice-Sophia Antipolis, Parc Valrose, 06108 Nice, France; E-Mail: gentajouve@unice.fr

**Keywords:** natural products, electronic circular dichroism, mass spectroscopy, cladocroic acid, sponge

## Abstract

The marine sponge *Haliclona fulva* (previously described as *Reniera fulva*) is widespread in the Mediterranean Sea. The chemical study of the sponge led to the isolation and identification of a new compound: 3-*epi*-cladocroic acid (**1**) alongside the previously reported cladocroic acid (**2**) and some other known compounds previously isolated. The structure was fully determined on the basis of extensive analysis by 1D and 2D NMR, as well as GC-MS/MS. The absolute configuration was determined by comparison of the experimental electronic circular dichroism (ECD) spectra with theoretically calculated spectra; these results may be extended to other asymetric cyclopropane carboxylic acids.

## 1. Introduction

*Haliclona (Halichoclona) fulva* (Demospongiae, Haplosclerida, Chalinidae), previously described as *Reniera fulva*, is an orange encrusting sponge, very common in the Mediterranean Sea [1]. The first chemical study of this sponge was performed by Cimino *et al.* and resulted in the isolation of six acetylenic compounds belonging to the renierin family [2]. Some years later, fulvinol, a more complex acetylenic compound, was characterized [3]. Surprisingly, terpenes usually found in *Haliclona mucosa*, have also been found in *H. fulva* samples [4]. During the past three decades, numerous alkaloids, such as sarains, haliclamines or manzamines, have been isolated from sponges of the genus *Haliclona* [5,6,7,8]. Even if alkaloids represent the largest chemical family of compounds isolated from this genus, almost all the families of natural products, including terpenoids, steroids, polyketides, fatty acids and peptides, have been found in species of this genus [9,10,11,12]. The huge chemodiversity found in *Halicona*’s specimens, and the change in genus of this sponge dramatically increased the interest in it. The most recent study reported the isolation of fulvynes, polyoxygenated acetylenic compounds related to osirisynes isolated from *Haliclona osiris* [13,14].

**Figure 1:**
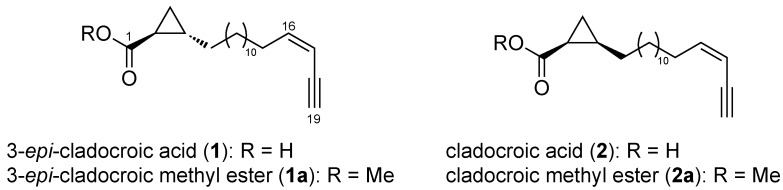
Structure of clodocroic acids **1, 2** and their methyl esters **1a, 2a**.

In our ongoing research on the chemodiversity present in Mediterranean sponges within the ECIMAR program (www.ecimar.org), we decided to undertake an additional chemical study of the sponge *H. fulva*. Our efforts were rewarded by the characterization of the 3-*epi*-cladocroic acid (**1**), a novel epimer of the cladocroic acid (**2**) (Figure 1), previously isolated from the sponge *Cladocroce incurvata* [15]. Unfortunately, during the first isolation of **2**, the determination of the absolute configuration suffered from the lack of experimental data, as only the specific optical rotation was reported. This usual pitfall in natural product chemistry has been overcome during the last years with the increasing use of chiroptical data such as electronic or vibrational circular dichroism (ECD and VCD) and theoretical calculations [16,17,18]. During the isolation process, renierin 1 , renierin 2, 18-dihydroreinirin 1 [2], fulvinol [3] and fulvynes A-I [13] were also identified.

Herein we report the complete structural characterization of **1** and **2** on the basis of 1D and 2D NMR and GC-MS/MS spectra. The absolute configurations of both compounds were determined by comparison of experimentally with theoretically calculated ECD spectra. Absolute configuration of the previously reported cladocroic acid was then addressed using the sign of the specific optical rotation of compound **2a** [15]. 

## 2. Experimental Section

### 2.1. General

Optical rotations were measured on a Jasco P-2000 polarimeter. UV and CD spectra were measured using a JASCO J-810 spectropolarimeter. NMR spectra were recorded at 25 °C on a Bruker Avance 500 spectrometer at 500/125 MHz (^1^H/^13^C). Chemical shifts were reported in ppm using residual CDCl_3_ (*δ* 7.26 for ^1^H and 77.2 for ^13^C) as internal reference. Coupling constants (*J*) are given in Hertz. HPLC purification was carried out on a Waters 600 system equipped with a Waters 717 plus autosampler, a Waters 996 photodiode array detector, and a Sedex 55 evaporative light-scattering detector (Sedere, France). GC-MS was performed on an Agilent technologies series 6890 GC coupled with the Agilent Technologies 5973 MSD. High resolution mass spectra (HRESIMS) were conducted on a LTQ Orbitrap mass spectrometer (Thermo Finnigan). 

### 2.2. Animal Material

One specimen of *Haliclona fulva* was collected by scuba diving along the coast of Saint-Jean-Cap-Ferrat (43° 41’ 29.9646”, 7° 19’ 11.8668”), at a depth of *ca*. 25 m. 

### 2.3. Extraction and Purification

After extraction of the freeze-dried sponge (50g) by a mixture of methanol and dichloromethane at a ratio of 1:1, the solvent was evaporated under reduced pressure yielding to 13.8g of crude extract. This extract was then fractionated by RP-C_18_ Vacuum Liquid Chromatography (elution with a decreasing polarity gradient of H_2_O/MeOH from 1:0 to 0:1, then MeOH/CH_2_Cl_2_ from 1:0 to 0:1). After an HPLC analysis of the five fractions previously obtained, we realized a further fractionation by Flash chromatography on a diol phase using a gradient of cyclohexane/EtOAc/MeOH (from 100% cyclohexane to 100% MeOH). Asubsequent HPLC purification of the cyclohexane/EtOAc/MeOH 3:1:0 fraction was realized usinga Symmetry C_18_ (7.8 × 300 mm;7 *µ*m) Waters™ column with a gradient of H_2_O/MeOH (from H_2_O/MeOH 50:50 to 0:100 in 16 min, flow rate = 3.0 mL.min^−1^) and afforded compounds **1** and **2** (1 mg). Separation of both compounds **1** and **2** was performed after methyl esterification using the same HPLC conditions. 

*3-*epi*-cladocroic acid* (**1**): ^1^H NMR (500 MHz) and ^13^C (125 MHz) NMR (CDCl_3_), see Table 1; 

*cladocroic acid* (**2**): ^1^H NMR (500 MHz) and ^13^C (125 MHz) NMR (CDCl_3_), see Table 1; 

**Table 1:** ^1^H and ^13^C NMR spectroscopic data (CDCl_3_, *δ* in ppm) of **1** and **2**.

	1		2	
Position	*δ_H_* (m, *J* Hz)	*δ_C_*	*δ_H_* (m, *J* Hz)	*δ_C_*
1	-	178.8	-	177.1^*a*^
2	1.35 (m, 1H)	19.8	1.69 (ddd, *J* = 8.8, 7.9, 5.4 Hz, 1H)	17.7
3	1.42 (m, 1H)	24.1	1.30 (m, 1H)	23.1
4	1.30 (m, 1H)	33.2	1.54 (m, 1H)	27.1
5-13	1.26 (m, 2H)	29.3-29.8	1.26 (m, 2H)	29.3-29.8
14	1.41 (m, 2H)	29.2	1.41 (m, 2H)	29.2
15	2.32 (q, *J* = 7.6 Hz, 2H)	30.4	2.32 (q, *J* = 7.6 Hz, 2H)	30.4
16	6.00 (dt, *J* = 10.4, 7.3 Hz, 1H)	146.5	6.00 (dt, *J* = 10.4, 7.3 Hz, 1H)	146.5
17	5.44 (ddd, *J* = 10.9, 3.5, 1.4 Hz, 1H)	108.1	5.44 (ddd, *J* = 10.9, 3.5, 1.4 Hz, 1H)	108.1
18	-	80.8	-	80.8
19	3.07 (d, *J* = 2.3 Hz, 1H)	81.3	3.07 (d, *J* = 2.3 Hz, 1H)	81.3
20a	0.78 (ddd, *J* = 8.0, 6.5, 4.1 Hz, 1H)	16.5	0.96 (dt, *J* = 10.2, 7.1, 5.2 Hz, 1H)	14.5
20b	1.22 (m, 1H)	16.5	1.08 (ddd, *J* = 8.5, 8.0, 4.6 Hz, 1H)	14.5

^*a*^ based on the HMBC spectrum.

### 2.4. Methyl Esterification of ***1*** and ***2***

The mixture of **1** and **2** (3 *µ*mol) was taken in a micro reaction vessel. Freshly distilled chlorotrimethylsilane (6 *µ*mol) was added slowly and stirred with a magnetic stirrer. Methanol (2 mL) was added to the reaction, and the resulting solution was stirred at room temperature until completion (reaction was monitored by TLC). The mixture was then evaporated under reduced pressure to obtain the products methyl ester **1a** and **2a**. 

*3-*epi*-cladocroic methyl ester* (**1a**): colorless solid; 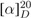 = + 21.5 (MeOH, *c* 0.1); UV (MeOH) *λ_max_* (log *ε*) 210 (0.34); CD (MeOH, c 3.15× 10^−4^ M) ∆*ε* (*λ_max_* nm) 0.81 (212); ^1^H NMR (500 MHz) NMR (CDCl_3_), see Table 2; EIMS *m*/*z* 318 M^+•^; HRESIMS *m*/*z* 341.24362 [M+Na]^+^; *calcd* for C_21_H_34_O_2_Na, 341.24510, ∆ -4.33618 ppm).

*cladocroic methyl ester* (**2a**): colorless solid; 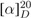 = + 12.1 (MeOH, *c* 0.1); UV (MeOH) *λ_max_* (log *ε*) 210 (0.54); CD (MeOH, c 3.15× 10^−4^ M) ∆*ε* (*λ_max_* nm) 0.32 (212); ^1^H NMR (500 MHz) NMR (CDCl_3_), see Table 2; EIMS *m*/*z* 318 M^+•^; HRESIMS *m*/*z* 341.24353 [M+Na]^+^; *calcd* for C_21_H_34_O_2_Na, 341.24510, ∆ -4.60447 ppm). 

**Table 2:** ^1^H NMR spectroscopic data (CDCl_3_, *δ* in ppm) of **1a** and **2a**.

	1a	2a
Position	*δ_H_* (m, *J* Hz)	*δ_H_* (m, *J* Hz)
1	-	-
2	1.35 (m, 1H)	1.68 (m, 1H)
3	1.39 (m, 1H)	1.30 (m, 1H)
4	1.30 (m, 1H)	1.66 (m, 1H)
5-13	1.25 (m, 2H)	1.25 (m, 2H)
14	1.31 (m, 2H)	1.30 (m, 2H)
15	2.32 (q, *J* = 7.6 Hz, 2H)	2.32 (q, *J* = 7.6 Hz, 2H)
16	6.00 (dt, *J* = 10.4, 7.3 Hz, 1H)	6.00 (dt, *J* = 10.4, 7.3 Hz, 1H)
17	5.44 (ddd, *J* = 10.9, 3.5, 1.4 Hz, 1H)	5.44 (ddd, *J* = 10.9, 3.5, 1.4 Hz, 1H)
18	-	-
19	3.07 (d, *J* = 2.3 Hz, 1H)	3.07 (d, *J* = 2.3 Hz, 1H)
20a	0.69 (ddd, *J* = 7.5, 6.7, 4.0 Hz, 1H)	0.96 (dt, *J* = 9.9, 7.1, 4.7 Hz, 1H)
20b	1.14 (ddd, *J* = 8.1, 5.0, 4.2 Hz, 1H)	1.01 (ddd, *J* = 11.8, 8.0, 4.5 Hz, 1H)
Me	3.67 (s, 3H)	3.67 (s, 3H)

### 2.5. Gas Chromatography Analysis

GC-MS analysis was conducted using an Agilent technologies series 6890 GC coupled with the Agilent Technologies 5973 MSD by injecting 0.5 µL with a 1/80 split on a Varian VF1-MS column (60 m × 0.25 mm × 0.25 *µ*m). The temperature of the injector was set to 270 °C. A temperature gradient of 5 °C.min^−1^ was applied to the column, starting from 60 °C to 270 °C. Helium was used as carrier gas (flow rate = 1 mL.min^−1^). The mass spectral detection was achieved using the quadrupole detector with an electronic ionization at 70 eV. 

### 2.6. Computational Details

**Figure 2:**
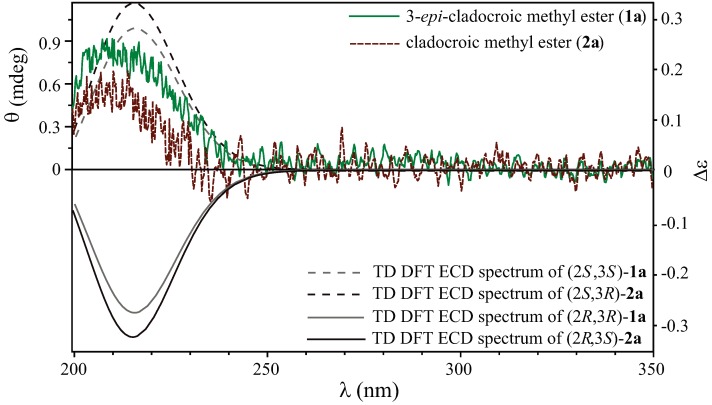
Experimental ECD spectra of compounds **1a** and **2a** with the theoretical TD-DFT calculated spectra of both enantiomers of compounds **1a** and **2a** without the alkyl enyne terminus chain.

Conformational analysis was performed using the conformer research algorithm implemented in the Conflex-Barista software [19,20,21,22]. All the Density Functional Theory (DFT) calculations were performed using the Gaussian 03 program package [23] at the B3LYP/6-311+G* level [24] using the integral equation formalism variant (IEFPCM) [25]. TD-DFT was employed to calculate excitation energy (in eV) and rotatory strength *R* in dipole velocity (*R_vel_*) and dipole length (*R_len_*) forms on the most stable conformers (∆*E* < 2 kcal.mol^−1^). The calculated rotatory strengths were simulated in ECD curve by using the corrected Gaussian function [26]:

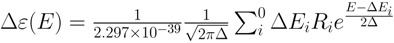

where ∆ is half the bandwidth at 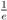 peak height and ∆*E* and *R* are the excitation energies and the rotatory strengths for transition *i* , respectively. For the Figure 2, *R_vel_* was used. 

## 3. Results and Discussion

### 3.1. Structure Elucidation

Compounds **1** and **2** were obtained as a colorless mixture, and determination of the planar structures was realized without any further purification step. ^1^H NMR signal at *δ* 1.26 ppm indicated that compounds **1** and **2** share a long alkyl chain (see Table 1). The signal at *δ* 81.3 (C, C-18) ppm on the ^13^C NMR spectrum, together with a thin doublet at *δ* 3.07 (d, *J* = 2.3 Hz, 1H, H-19) ppm on the ^1^H NMR spectrum, were attributed to an acetylenic proton. COSY correlations between this acetylenic proton and ethylenic protons at *δ* 5.44 (ddd, *J* = 10.9, 3.5, 1.4 Hz, 1H, H-17) and *δ* 6.00 (dt, *J* = 10.4, 7.3 Hz, 1H, H-16) ppm evidenced a *Z* enyne terminus substituted by three spin-coupled methylenes at *δ* 2.32 (q, *J* = 7.6 Hz, 2H, H-15), 1.41 (m, 2H, H-14) and 1.26 (m, 2H, H-13) ppm. The *Z* configuration of the C=C double bond was confirmed by the shielding of the the adjacent vinylic methylene at *δ* 30.4 (CH_2_, C-15) ppm. Downfield shifted signals (> 35 ppm) are usually observed for an *E* configuration [27]. 

Inspection of the ^1^H NMR spectrum also evidenced characteristic cyclopropane signals at *δ* 0.78 (ddd, *J* = 8.0, 6.5, 4.1 Hz, 1H, H-20a), 0.96 (dt, *J* = 10.2, 7.1, 5.2Hz, 1H, H-20a) and 1.08 (ddd, *J* = 8.5, 8.0, 4.6 Hz, 1H, H-20b) and 1.22 (m, 1H, H-20b) ppm. Indeed an important delocalisation of the *σ* electrons in a cyclopropane can exert a considerable influence on the chemical shifts of neighbouring protons. The resulting ^1^H chemical shifts are lower than those observed for usual methylenes [28]. 

Analysis of the HMBC spectrum gave supplementary information on the structure. HMBC correlations H-16/C-18, H-16/C-17 and H-16/C-15 confirmed the position of the enyne terminus. Another HMBC correlation was observed between the non equivalent protons of the cyclopropane ring H-20a and H-20b with the C-4 connecting the ring with the rest of the molecules. Although two carboxylic acid were identified on the HMBC spectrum, the absence at 2.2 ppm in the ^1^H NMR spectrum of the characteristic triplet signal of a methylene adjacent to a carbonyl, suggested that the cyclopropane ring was alpha connected to the carboxylic acid function. This conclusion was supported by the HMBC correlations H-20a/C-1 and H-20b/C-1. After anlysis of the HMBC and COSY spectra, two sets of correlations were identified for two distinct cyclopropane rings, but no correlations where observed to link them together. We came to the conclusion that two diastereoisomers of the cyclopropane ring (*cis* and *trans*) were present in the mixture. A comparison with ^1^H and ^13^C NMR data obtained by dAuria *et al.* for cladocroic acid (**1**) was consistent with this conclusion [15]. Indeed, in the previously published paper, only two signals for the cyclopropane ring were reported at *δ* 0.96 and 1.08 ppm while four signals were observed on the ^1^H NMR spectrum at *δ* 0.78, 0.96, 1.08 and 1.22 ppm in our case. 

To separate both compounds **1** and **2**, a methyl esterification was realized in order to increase the hydrophobic interactions of the compounds with the reverse phase column [29]. A mixture of the two methyl ester derivatives was obtained and subsequently purified using the same conditions than those previously described. ^1^H NMR spectra of collected peaks were recorded to confirm that both compounds were successfully separated (Table 2). 

Finally, the length of the methyl ester derivatives alkyl chain was reliably determined by GC-MS. A fairly similar fragmentation pattern and the same molecular ion at *m*/*z* 318 were observed for compounds **1a** and **2b**. Identification of the fragments led to the conclusion that the alkyl chain contained 12 methylenes (Figure S11). 

Comparing NMR data and the signs of the optical rotation, we concluded that compound **2a** was cladocroic acid previously isolated from *C. invurvata*. Since the authors were unable to assign the absolute configuration [15], compound **2** can be first described as (2S^*^, 3R^*^)-cladocroic acid while compound **1** can be identified as (2S^*^,3S^*^)-cladocroic acid. To determine the absolute configuration of both compounds, we then decided to use ECD, a nondestructive and very sensitive method that has been increasingly employed in the last few years in natural product chemistry [16,17,18]. 

### 3.2. Determination of the Absolute Configuration

To take advantage of the presence of the ester function chromophore close to the chiral centers of the two compounds **1a** and **2a**, a study of the absolute configuration was performed by ECD. The double bond of the ester implied two possible electronic transitions, the allowed *π* → π^*^ transition that gives a high *ε* value, and the forbidden *n* → π^*^ transition which has a far less important *ε* value [30]. Since the *π* → π^*^ transition has a *λ_max_* = 185 nm that falls below the measurement window (> 200 nm), the study was realized on the *n* → π^*^ forbidden transition whereby the energy level is lower (*λ_max_* = 205 nm). The linear methylenes induced a high degree of free rotation during the conformational analysis; the theoretical calculations were performed on a simplified analog (*i.e*., the cyclopropane substituted by the ester and an ethyl chain). This approximation was considered to be realistic as the effects of the enyne terminus were considered negligible. 

Only one Cotton effect (CE) was observed on the ECD spectrum at *λ* = 210 nm, and the difference of 5 nm between the value and the experimental observation was explained by a slight bathochrome effect of the aliphatic chain (Woodward-Fieser rules). TD-DFT calculations using the integral equation formalism variant (IEFPCM) have been performed on the most stable conformer to simulate the ECD spectrum of both compounds **1a** and **2a** (Figure 2). 

A simple comparison of the sign of the CE at *ca*. 210 nm indicated that both compounds shared the same absolute configuration on C-2 and could be describe as (2*S*,3*S*)-*epi*-cladocroic methyl ester (**1a**) and (2*S*,3*R*)-cladocroic methyl ester (**2a**). Since the methyl esterification did not change the absolute configuration of **1** and **2**, these results also provided information on the absolute configuration of the previously isolated cladocroic acid (**2**), which also had the same *S* configuration at C-2. 

## 4. Conclusions

We report here the isolation of 3-*epi*-cladocroic acid (**1**) from the Mediterranean sponge *Haliclona fulva*, a new epimer of the known cladocroic acid (**2**) previously isolated from the marine sponge *Cladocroce incurvata*. The structural elucidation was conducted using extensive 1D and 2D NMR data analysis, in addition to GC-MS/MS. The absolute configuration of both compounds **1** and **2** was addressed by comparison of the sign of the Cotton effect observed on the experimentally and theoretically calculated ECD spectra acquired on their methyl ester derivatives **1a** and **2a**. 

## References

[1] Van Soest R., Boury-Esnault N., Hooper J., Rützler K., de Voogd N., Alvarez de Glasby B., Hajdu E., Pisera A., Manconi R., Schoenberg C. (2011). World Porifera Database. http://www.marinespecies.org/.

[2] Cimino G., de Stefano S. (1977). New acetylenic compounds from the sponge *Reniera fulva*. Tetrahedron Lett..

[3] Ortega M.J., Zubía E., Carballo J.L., Salvá J. (1996). Fulvinol, a new long-chain diacetylenic metabolite from the sponge *Reniera fulva*. J. Nat. Prod..

[4] Zubía E., Ortega M.J., Carballo J.L., Salvá J. (1994). Sesquiterpene hydroquinones from the sponge *Reniera mucosa*. Tetrahedron.

[5] Defant A., Mancini I., Raspor L., Guella G., Turk T., Sepčić K. (2011). New structural insights into saraines A, B, and C, macrocyclic alkaloids from the mediterranean sponge *Reniera* (*Haliclona*) *sarai*. Eur. J. Org. Chem..

[6] Jang K.H., Kang G.W., Jeon J.E., Lim C., Lee H.S., Sim C.J., Oh K.B., Shin J. (2009). Haliclonin a, a new macrocyclic diamide from the sponge *Haliclona* sp. Org. Lett..

[7] Timm C., Mordhorst T., Köck M. (2010). Synthesis of 3-alkyl pyridinium alkaloids from the arctic sponge *Haliclona viscosa*. Mar. Drugs.

[8] Sakai R., Higa T., Jefford C.W., Bernardinelli G. (1986). Manzamine a, a novel antitumor alkaloid from a sponge. J. Am. Chem. Soc..

[9] Barrow R., Capon R. (1991). Alkyl and alkenyl resorcinols from an australian marine sponge, *Haliclona* sp. (haplosclerida : Haliclonidae). Aust. J. Chem..

[10] Sheikh Y.M., Djerassi C. (1974). Steroids from sponges. Tetrahedron.

[11] Wang G.Y.S., Abrell L.M., Avelar A., Borgeson B.M., Crews P. (1998). New hirsutane based sesquiterpenes from salt water cultures of a marine sponge-derived fungus and the terrestrial fungus *Coriolus consors*. Tetrahedron.

[12] Rashid M.A., Gustafson K.R., Boswell J.L., Boyd M.R. (2000). Haligramides a and b, two new cytotoxic hexapeptides from the marine sponge *Haliclona nigra*. J. Nat. Prod..

[13] Nuzzo G., Ciavatta M.L., Villani G., Manzo E., Zanfardino A., Varcamonti M., Gavagnin M. (2012). Fulvynes, antimicrobial polyoxygenated acetylenes from the mediterranean sponge *Haliclona fulva*. Tetrahedron.

[14] Shin J., Seo Y., Cho K.W., Rho J.R., Paul V.J. (1998). Osirisynes A-F, highly oxygenated polyacetylenes from the sponge *Haliclona osiris*. Tetrahedron.

[15] D’Auria M.V., Paloma L.G., Minale L., Riccio R., Zampella A., Debitus C. (1993). Metabolites of the new caledonian sponge *Claodocroce incurvata*. J. Nat. Prod..

[16] Diedrich C., Grimme S. (2003). Systematic investigation of modern quantum chemical methods to predict electronic circular dichroism spectra. J. Phys. Chem. A.

[17] Laville R., Genta-Jouve G., Urda C., Fernández R., Thomas O.P., Reyes F., Amade P. (2009). Njaoaminiums A, B, and C: Cyclic 3-alkylpyridinium salts from the marine sponge *Reniera* sp. Molecules.

[18] Pescitelli G., Kurtán T., Krohn K. (2012). Assignment of the Absolute Configurations of Natural Products by Means of Solid-State Electronic Circular Dichroism and Quantum Mechanical Calculations. Comprehensive Chiroptical Spectroscopy.

[19] Goto H., Osawa E. (1989). Corner flapping: A simple and fast algorithm for exhaustive generation of ring conformations. J. Am. Chem. Soc..

[20] Goto H., Osawa E. (1992). Further developments in the algorithm for generating cyclic conformers. Test with cycloheptadecane. Tetrahedron.

[21] Goto H., Osawa E. (1993). An efficient algorithm for searching low-energy conformers of cyclic and acyclic molecules. J. Chem. Soc. Perkin Trans. 2.

[22] Goto H., Ohta K., Kamakura T., Obata S., Nakayama N., Matsumoto T., Osawa E.  (2004). CONFLEX 6.

[23] Frisch M.J., Trucks G.W., Schlegel H.B., Scuseria G.E., Robb M.A., Cheeseman J.R., Montgomery J.A., Vreven T., Kudin K.N., Burant J.C. (2004). Gaussian 03, Revision C.01.

[24] Lee C., Yang W., Parr R.G. (1988). Development of the colle-salvetti correlation-energy formula into a functional of the electron density. Phys. Rev. B.

[25] Tomasi J., Mennucci B., Cammi R. (2005). Quantum mechanical continuum solvation models. Chem. Rev..

[26] Stephens P.J., Harada N. (2010). ECD cotton effect approximated by the gaussian curve and other methods. Chirality.

[27] Wang G.Y.S., Kuramoto M., Uemura D., Yamada A., Yamaguchi K., Yazawa K. (1996). Three novel anti-microfouling nitroalkyl pyridine alkaloids from the okinawan marine sponge *Callyspongia* sp. Tetrahedron Lett..

[28] Poulter C.D., Boikess R.S., Brauman J.I., Winstein S. (1972). Shielding effects of a cyclopropane ring. J. Am. Chem. Soc..

[29] Li J., Sha Y. (2008). A convenient synthesis of amino acid methyl esters. Molecules.

[30] Henderson G. (1990). A new look at carbonyl electronic transitions. J. Chem. Educ..

